# Linear high-dimensional mediation models adjusting for confounders using propensity score method

**DOI:** 10.3389/fgene.2022.961148

**Published:** 2022-10-10

**Authors:** Linghao Luo, Yuting Yan, Yidan Cui, Xin Yuan, Zhangsheng Yu

**Affiliations:** ^1^ Department of Bioinformatics and Biostatistics, School of Life Sciences and Biotechnology, Shanghai Jiao Tong University, Shanghai, China; ^2^ SJTU-Yale Joint Center for Biostatistics, Shanghai Jiao Tong University, Shanghai, China; ^3^ Jinmai Community Service Center, Guiyang, China; ^4^ Clinical Research Institute, Shanghai Jiao Tong University School of Medicine, Shanghai, China

**Keywords:** high-dimensional mediation model, confounders, propensity score, inverse probability weighting, SIS, MCP, joint-significance test

## Abstract

High-dimensional mediation analysis has been developed to study whether epigenetic phenotype in a high-dimensional data form would mediate the causal pathway of exposure to disease. However, most existing models are designed based on the assumption that there are no confounders between the exposure, the mediators, and the outcome. In practice, this assumption may not be feasible since high-dimensional mediation analysis (HIMA) tends to be observational where a randomized controlled trial (RCT) cannot be conducted for some economic or ethical reasons. Thus, to deal with the confounders in HIMA cases, we proposed three propensity score-related approaches named PSR (propensity score regression), PSW (propensity score weighting), and PSU (propensity score union) to adjust for the confounder bias in HIMA, and compared them with the traditional covariate regression method. The procedures mainly include four parts: calculating the propensity score, sure independence screening, MCP (minimax concave penalty) variable selection, and joint-significance testing. Simulation results show that the PSU model is the most recommended. Applying our models to the TCGA lung cancer dataset, we find that smoking may lead to lung disease through the mediation effect of some specific DNA-methylation sites, including site Cg24480765 in gene RP11-347H15.2 and site Cg22051776 in gene KLF3.

## 1 Introduction

Mediation analysis was proposed by Baron and Kenny (1986). It has been widely used in sociological, psychological, and medical research (MacKinnon et al., 2007; Lockhart et al., 2011; Wen and Ye, 2014), aiming to study how a primary exposure X indirectly affects the outcome Y through one or more mediators M (MacKinnon et al., 2007). For instance, epigenetic marks ( M ) such as DNA methylation are believed to mediate the causal pathway of smoking ( X ) to disease occurrence ( Y ) (Cortessis et al., 2012; Valeri et al., 2017; Fujii et al., 2021). Notably, due to the advancement in high throughput technology, epigenetic data are usually generated in a high-dimensional form. The need for mediation analysis toward high-dimensional epigenetic data motivates mediation analysis to be developed from low to high dimensions. Many scholars have focused on the hypothesis testing method under high-dimension cases (Huang and Pan, 2016; Djordjilovic et al., 2019; Gao et al., 2019); while for the mediator selection problem, Zhang et al. first proposed a complete high-dimensional mediation analysis (HIMA) model based on SIS dimension reduction, MCP penalty estimation, and joint-significance test (Zhang et al., 2016). Furthermore, HIMA was generalized to survival outcome and non-linear assumptions for different application scenarios (Loh et al., 2020; Luo et al., 2020; Cui et al., 2021; Zhang et al., 2021).

Nevertheless, the premise of an unbiased inference in mediation analysis is the no-confounding assumption: there are no confounders between the exposure, the mediators, and the outcome (VanderWeele, 2009). Imai et al. (2010) modified it as a sequential ignorability assumption: 1) given the confounders, the treatment assignment is assumed to be ignorable (independent of outcomes and mediators); 2) given the confounders and exposure, the mediator is ignorable. Part 1) can be satisfied by RCT, while part 2) is often considered to be irrefutable (Manski, 2007), which is hard to guarantee even in RCT. Thus, in this study, we assume by default that part 2) holds and mainly focus on the confounding problem caused by non-randomization. In most high-dimensional mediation cases, RCT is not feasible because of the economic cost or ethical issues. This results in an uneven distribution of confounders between exposure groups. For example, when exploring the relationship among smoking X , DNA methylation M and disease occurrence Y , the baseline factors such as age and gender would also have an impact on smoking status and disease occurrence (e.g., males may be more likely to smoke and more vulnerable to lung disease than females, and we cannot force non-smokers to be smokers). Moreover, the baseline factors tend to be unevenly distributed in the smoking group and the non-smoking group because of the non-randomization. Thus, the confounding problem is almost inevitable.

To adjust for the confounders in observational studies, regression analyses (e.g., linear and logistic regression) are the most popular due to their simplicity (Normand et al., 2005). Nonetheless, when there are a large number of variables, regression may work inefficiently and another helpful tool, propensity score (PS), would be more powerful (Lu, 2009). A propensity score represents the probability for an individual to have been assigned to an exposure (or treatment), conditional on a host of potential confounders (Lanza et al., 2013). By controlling the propensity score in a proper way like matching, regression, or inverse probability weighting, the confounders could be adjusted, which helps to create a theoretical randomized controlled trial (RCT) (Rosenbaum and Rubin, 1983; D'Agostino, 1998) and satisfy the ignorability assumption. Compared with the regression adjustments, propensity score concentrates all covariates into a single “score” variable, which is more flexible and adequate to eliminate confounding bias (Austin, 2011; Yu et al., 2021). Previous studies have already applied PS in mediation analysis (Coffman, 2011; Jo et al., 2011; Yu et al., 2021). However, there is still a lack of insights into the appropriate utilization of PS for adjusting confounders in HIMA under continuous (or binary) outcomes.

Therefore, in this article, we proposed three propensity score-related approaches to adjust for confounders in HIMA with continuous outcomes. The first two methods are inspired, respectively, by viewing PS as a covariate or using PS to conduct weighted estimation. The third method is a hybrid of the former two. Our results show that the hybrid model performs the best, with the most accurate inference result.

The structure of this article is as follows. The following section introduces the proposed high-dimensional mediation models, adjusting for confounders based on the propensity score. Then, we show the simulation results to illustrate the performance of the models. Additionally, we apply our models to the lung dataset in TCGA, identifying the true DNA methylation sites that mediate the causal pathway of smoking in lung disease. Lastly, we summarize and list prospects of future research.

## 2 Methods

### 2.1 The model

In typical observational HIMA research with a sample size of n , we define the exposure variable as X , where X=1 represents the treatment group and X=0 represents the controlled group; let Y be the continuous outcome variable, $M={\left(M_{1},M_{2},\ldots ,M_{p}\right)}^{T}$ be the p− dimensional (p≫n) potential mediators, and $Z={\left(Z_{1},Z_{2},\ldots ,Z_{w}\right)}^{T}$ be the baseline confounders. For individual i,i=1,2,3,…,n , we have the model:

$$\begin{array}{c} M_{ki}=c_{k}+\alpha_{k}X_{i}+\Theta_{k}^{T}Z_{i}+e_{ki}\;,\;k=1,2,\ldots p\;\\ Y_{i}=c+\gamma X_{i}+\beta^{T}M_{i}+\Phi^{T}Z_{i}+\xi_{ki} \end{array}.$$ (1)

**FIGURE 1:**
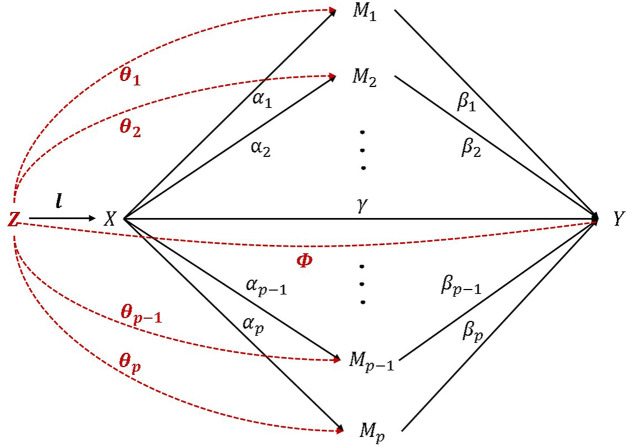
High-dimensional mediation model with confounders.

Note that $\alpha ={\left(\alpha_{1},\ldots ,\alpha_{p}\right)}^{T}$ is the coefficient vector from exposure X to mediators $M={\left(M_{1},\ldots ,M_{p}\right)}^{T}$ , while $\beta ={\left(\beta_{1},\ldots ,\beta_{p}\right)}^{T}$ is the coefficient vector from $M={\left(M_{1},\ldots ,M_{p}\right)}^{T}$ to outcome Y ; $\alpha_{k}\beta_{k}$ corresponds to the mediation effect of $M_{k}$ . If $\alpha_{k}\beta_{k}\neq 0$ , we consider $M_{k}$ as a significant mediator; $\Phi ={\left(\varphi_{1},\varphi_{2},\ldots ,\varphi_{w}\right)}^{T}$ is the coefficient vector measuring the effect of Z on Y ; $\Theta_{k}={\left(\theta_{k1},\theta_{k2},\ldots ,\theta_{kw}\right)}^{T}$ relates to the effect of confounders Z on mediator $M_{k}$ . The relationship between variables in the model is shown in Figure 1:

### 2.2 Methodology

#### 2.2.1 Adjusting confounders using propensity score

Since there are baseline confounders, we integrate a propensity score (PS) into the model. Rosenbaum and Rubin (Rubin, 1983) defined propensity score as the probability of treatment assignment according to the baseline covariates $Z={\left(Z_{1},\ldots ,Z_{m}\right)}^{T}$ :

$$\begin{array}{c} S=P\left(X=1|Z_{1},\ldots ,Z_{m}\right) \end{array}.$$

The propensity score represents the probability of an individual i, i=1,2,…n being allocated to the treatment group X=1 . In practice, the application procedure can be summarized as follows: first, estimate the propensity score and then adopt various methods such as matching, regression, weighting, *etc.*, to adjust for confounding. Finally, evaluate the adjusted causal effect. The propensity score can be evaluated by logistic regression (Lanza et al., 2013):

$$\begin{array}{c} logit\left(S_{i}=P\left(X_{i}=1\right)\right)=b_{0}+b_{1}Z_{1i}+\ldots +b_{m}Z_{mi} \end{array}.$$

In consideration of the baseline confounders, the actual high-dimensional mediation analysis model is shown in (1). Therefore, we adopt propensity score regression (PSR) and propensity score weighting (PSW) to reduce the bias.

The main idea of PSR is adding the PS variable into regression. The propensity score can be regarded as the “coarsest function” of the confounding covariates (D’Agostino, 1998). Therefore, controlling the propensity score in regression works similar to taking all the confounders as covariates when estimating. We can use a linear regression model, if the outcome variable is continuous, and a logistic regression model, if the outcome variable is binary (Austin, 2011). We estimate the effect of the model (2):

$$\begin{array}{c} M_{ki}=c_{k}+{\hat{\alpha }}_{k}X_{i}+\hat{k}S_{i}+e_{ki}\;,\;k=1,2,\ldots p\;\\ Y_{i}=c+\hat{\gamma }X_{i}+{\hat{\beta }}^{T}M_{i}+\hat{k}S_{i}+\xi_{ki}. \end{array}$$ (2)

In contrast, PSW first constructs inverse probability weights from the propensity score for individual i (Hirano and Imbens, 2001):

$$\begin{array}{c} w_{i}=\frac{X_{i}}{S_{i}}+\frac{\left(1-X_{i}\right)}{\left(1-S_{i}\right)} \end{array}.$$

The weighted sample satisfies the condition that exposure (or treatment) assignment is independent of the baseline covariates (Austin and Stuart, 2015), and meets the ignorability assumption. Consequently, by weighted estimation, we can get an unbiased estimation of the coefficient related to X :

$$\begin{array}{c} M_{ki}=c_{k}+c_{z}+{\hat{\alpha }}_{k,w}X_{i}+e_{ki}\;,\;k=1,2,\ldots p\\ Y_{i}=c+c_{z}^{*}+{\hat{\gamma }}_{k,w}X_{i}+{\hat{\beta }}^{T}M_{i}+\xi_{ki}. \end{array}$$ (3)

In the above formula, ${\hat{\alpha }}_{k,w}\;and\;{\hat{\gamma }}_{k,w}$ are the coefficients by weighted estimation according to the weight vector S .

In the preliminary Monte Carlo simulation, we found that PSM performs better in the estimation of $\alpha_{k}$ , while PSW works more efficiently in the $\beta_{k}$ selection. Therefore, we combine the two approaches by using PSM in the M mediator model component and using PSW in the Y outcome component. The new model is named PSU, as shown below:

$$\begin{array}{c} M_{ki}=c_{k}+{\hat{\alpha }}_{k}X_{i}+\hat{k}S_{i}+e_{ki}\;,\;k=1,2,\ldots p\\ Y_{i}=c+c_{z}^{*}+{\hat{\gamma }}_{k,w}X_{i}+{\hat{\beta }}^{T}M_{i}+\xi_{ki} \end{array}$$ (4)

We apply these three model ideas to steps 2–4 in the following procedure.

#### 2.2.2 Procedure

We take the analysis procedure used by Zhang et al. (2016) as HIMA and propose to use the propensity score to adjust for confounders in the HIMA procedure. The detailed procedure is as follows:

1. The propensity score and inverse probability weight were calculated.

First, X was taken as the response and Z as the predictors to fit the logical model, and the propensity score was calculated:

$$logit\left(S_{i}=P\left(X_{i}=1\right)\right)=l_{0}+l_{1}Z_{1i}+\ldots +l_{m}Z_{mi},$$

$$S_{i}=\frac{\exp\left(l_{0}+l_{1}Z_{1i}+\ldots +l_{m}Z_{mi}\right)}{1+\exp\left(l_{0}+l_{1}Z_{1i}+\ldots +l_{m}Z_{mi}\right)}.$$

Then, we calculated the weight. The weight of the group was given as X=1 as 1/ S , and that of the group X=0 as 1/(1−S ):

$$w_{i}=\frac{X_{i}}{S_{i}}+\frac{\left(1-X_{i}\right)}{\left(1-S_{i}\right)}.$$

2. The dimension was reduced by sure independence screening (SIS).

Penalty estimation methods such as MCP and SCAD may not perform ideally in accuracy and computational cost under an ultra-high-dimensional variable space (Fan and Lv, 2008). Thus, we first adopted the sure independence screening (SIS) (Fan and Lv, 2008) method to reduce dimension p from high-dimensional to a moderate scale $d=\left[\frac{2n}{\log\left(n\right)}\right]$ . The set $I_{SIS}$ was identified:

$$I_{SIS}=\left\{k:\;M_{k}\;is\;among\;the\;top\;d\;mediators\;with\;largest\;effect\;to\;Y\;\right\}.$$

For PSR and PSU methods, $\beta_{k}$ can be estimated by maximum likelihood estimation (MLE):

$$\partial \frac{\sum \mathrm{Log}\;\left({\;p}_{1}\left(Y_{i}\right)\;\right)}{\partial \beta_{k}}=0,$$

where the maximum likelihood function $p_{1}\left(Y_{i}\right)$ is:

$$p_{1}\left(Y_{i};c,\gamma ,\beta_{k},k\right)=\frac{1}{\sqrt{2\pi }}\exp\left\{-\frac{1}{2}{\left(Y_{i}-c-\gamma X_{i}-\beta_{k}M-kS\right)}^{2}\right\}.$$

For the PSW method, since the confounders indirectly affect $\beta_{k}$ by interfering with the coefficient γ , we adopt a “two step” weighting method. For each $M_{k}$ , ${\hat{\gamma }}_{k,w}$ is obtained by weighted MLE:

$$\partial \frac{\sum w_{i}\mathrm{Log}\left(\;p_{2}\left(Y_{i}\right)\;\right)\;}{\partial \beta_{k}}=0,$$

where the maximum likelihood function $p_{2}\left(Y_{i}\right)$ is:

$$p_{2}\left(Y_{i};c,\gamma ,\beta_{k}\right)=\frac{1}{\sqrt{2\pi }}\exp\left\{-\frac{1}{2}{\left(Y_{i}-c-\gamma X_{i}-\beta_{k}M\right)}^{2}\right\}.$$

After obtaining ${\hat{\gamma }}_{k,w}$ , the residual can be derived:

$${\hat{e}}_{k}=Y-{\hat{\gamma }}_{k,w}X.$$

Then $\beta_{k}$ can be simply acquired by fitting the regression model of $e_{k}∼M_{k}$ without considering weight.

The purpose of SIS is to filter out most of the mediators that are irrelevant or weakly related to the response.

3. Candidate mediators for testing through MCP-penalized estimation were selected.

Through SIS, we obtained a set of potential mediators with d -dimension:

$$M_{SIS}=\left\{M_{k}:\;k\in I_{SIS}\;\right\},$$

Then, we employed MCP-penalized estimation to further select mediators. For PSR method, we minimized the sum of squared residuals including propensity score term S :

$$\overset{n}{\underset{i=1}{\sum }}{\left(Y_{i}-c-\gamma X_{i}-\sum_{j\in I_{SIS}}\beta_{j}M_{ij}-S\right)}^{2}+\sum_{k\in I_{SIS}}p\left(\beta_{k}\right).$$

For PSW and PSU, we minimized the sum of squared residuals:

$$\overset{n}{\underset{i=1}{\sum }}{\left(Y_{i}-c-\gamma X_{i}-\sum_{j\in I_{SIS}}\beta_{j}M_{ij}\right)}^{2}+\sum_{k\in I_{SIS}}p\left(\beta_{k}\right).$$

We selected the MCP penalty function:

$$p\left(\beta_{k}\right)=\lambda \left[\left|\beta_{k}\right|-\frac{|\beta_{k}{|}^{2}}{2\delta \lambda }\right]I\left\{0\leq \left|\beta_{k}\right|<\delta \right\}+\frac{\lambda^{2}\delta }{2}I\left\{\left|\beta \right|\geq \delta \lambda \right\},$$

where λ is the regularization parameter, which can be selected by AIC and BIC; δ is the tuning parameter. According to Zhang (2010), MCP is preferred to other penalty functions because MCP can choose the correct model with a probability tending to 1, and the procedure can be acquired in the R package *ncvreg* presented by Breheny and Huang (2011).

4. Joint-significance test.

$M_{k}$ is considered a true mediator when ${\hat{\alpha }}_{k}$ and ${\hat{\beta }}_{k}$ are significant simultaneously. In other words, mediator $M_{k}$ will be identified if both the hypothesis $H_{0,\beta_{k}}:{\hat{\beta }}_{k}=0$ and $H_{0,\alpha_{k}}:{\hat{\alpha }}_{k}=0$ are rejected. Let $C=\left\{k:{\hat{\beta }}_{k}\neq 0\right\}$ represent the results based on the penalized estimation. Then, we performed the joint-significance test for the $M_{k}$ in set $C=\left\{k:{\hat{\beta }}_{k}\neq 0\right\}$ .

For $H_{0,\beta_{k}}:{\hat{\beta }}_{k}=0$ , the *p*-value can be obtained:

$$P_{raw,\beta_{k}}=2\left\{1-\Phi \left(\frac{\left|{\hat{\beta }}_{k}\right|}{{\hat{\sigma }}_{\beta_{k}}}\right)\right\},$$

where Φ(·) is the cumulative distribution function of the standard normal distribution N(0,1) ; ${\hat{\sigma }}_{k,\beta }$ is the estimated standard error of ${\hat{\beta }}_{k}$ which can be calculated through the oracle property of MCP. The obtained p -value was then corrected by the Benjamini–Hochberg (BH) method to control the false discovery rate (FDR). The $P_{raw,\beta_{k}}$ was ranked incrementally, and $r_{\beta_{k}}$ was assumed to be the location number of ${\hat{\beta }}_{k}$ , then $P_{BH,\beta_{k}}$ was:

$$P_{BH,\beta_{k}}=\max\left(P_{raw,\beta_{k}}\cdot \frac{p}{r_{\beta_{k}}},\;1\right).$$

Here, we chose to control FDR instead of family-wise error rate (FWER) because FDR gave a less conservative way than FWER to detect mediators in HIMA. Similarly, the p -value for $H_{0,\alpha_{k}}:{\hat{\alpha }}_{k}=0$ is:

$$P_{raw,\;\alpha_{k}}=2\left\{1-\Phi \left(\frac{\left|{\hat{\alpha }}_{k}\right|}{{\hat{\sigma }}_{\alpha_{k}}}\right)\right\}.$$

The effect ${\hat{\alpha }}_{k}$ is estimated by the first equation in model (2) for PSR and PSU and the first equation in model (3) for PSW. Also $P_{raw,\;\alpha_{k}}$ can be corrected by the BH method:

$$P_{BH,\;\alpha_{k}}=\max\left(P_{raw,\;\alpha_{k}\cdot }\frac{p}{r_{\alpha_{k}}},\;1\right).$$

Finally, the joint-significance p -value for $M_{k}$ is defined as the max one of $P_{BH,\alpha_{k}}$ and $P_{BH,\beta_{k}}$ :

$$P_{\begin{array}{c} M_{k}\\ \; \end{array}}=\max\;\left(P_{BH,\alpha_{k}}\;,P_{BH,\beta_{k}}\right).$$

**FIGURE 2:**
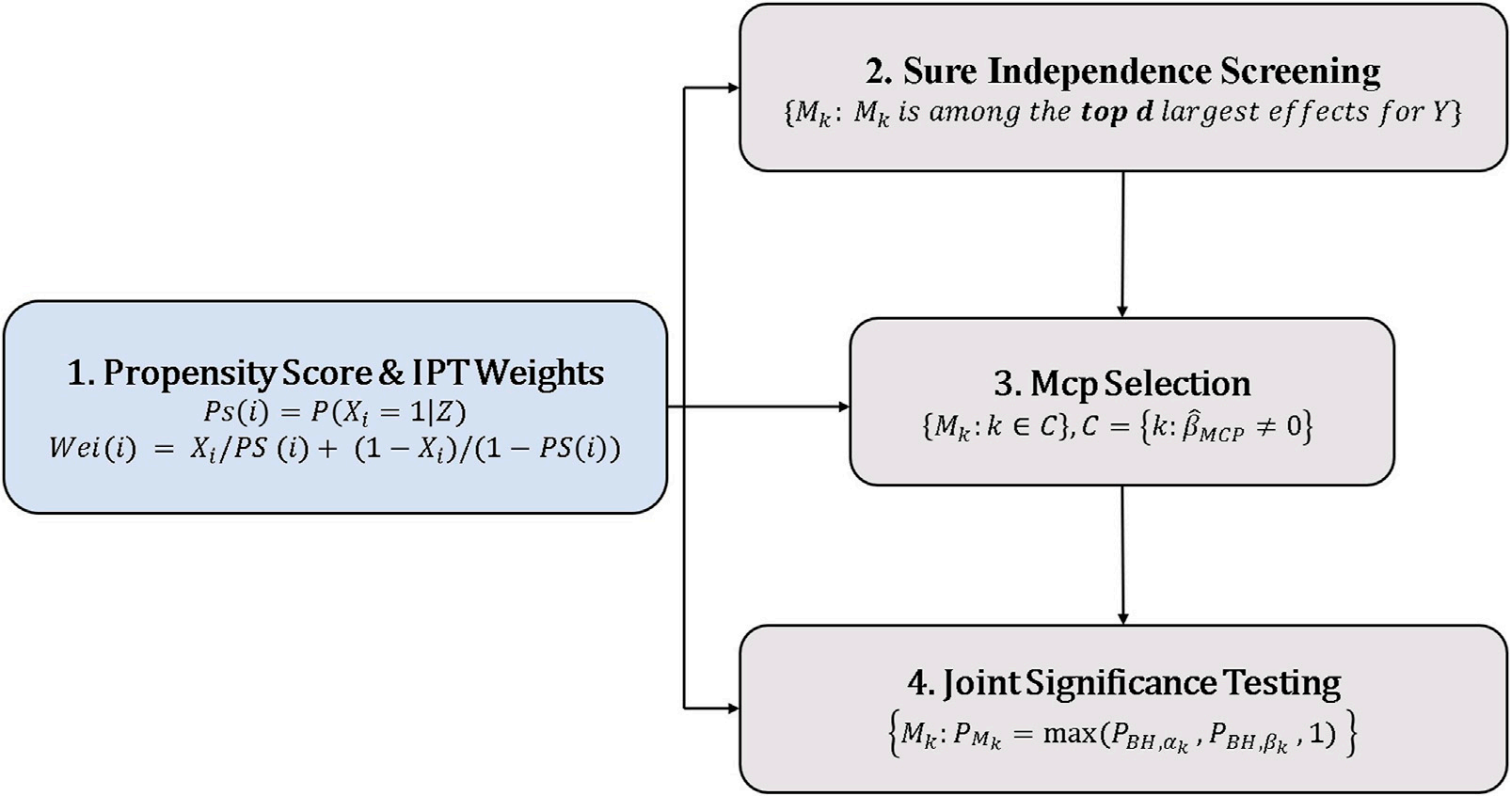
The structure of the high-dimensional mediation models with PS adjusting for confounders.

We set the type I error rate α as 0.05 for all the tests. The brief structure of the whole procedures is summarized in Figure 2.

### 3 Simulation

In this section, we will evaluate our models by simulation studies. The simulation data are generated according to the true model (1):

$$\begin{array}{c} M_{ki}=c_{k}+\alpha_{k}X_{i}+\Theta_{k}^{T}Z_{i}+e_{ki}\;,\;k=1,2,\ldots p\;\\ Y_{i}=c+\gamma X_{i}+\beta^{T}M_{i}+\Phi^{T}Z_{i}+\xi_{ki} \end{array}.$$

Ten confounders $Z={\left(Z_{1},\cdots ,Z_{10}\right)}^{T}$ between X , M , and Y are considered, of which $Z_{1}-Z_{5}$ follow independent Bernoulli distribution B(0.3) and $Z_{6}-Z_{10}$ follow multivariate normal distribution with a mean vector $\mu ={\left(0,0,0,0,0\right)}^{T}$ and a covariance matrix Σ :

$$\Sigma =\left[\begin{array}{cc} \begin{array}{cc} 1 & 0.3 \end{array} & \begin{array}{cc} 0.3 & \begin{array}{cc} 0.3 & 0.3 \end{array} \end{array}\\ \begin{array}{cc} 0.3 & 1\\ 0.3 & 0.3\\ \begin{array}{c} 0.3\\ 0.3 \end{array} & \begin{array}{c} 0.3\\ 0.3 \end{array} \end{array} & \begin{array}{cc} 0.3 & \begin{array}{cc} 0.3 & 0.3 \end{array}\\ 1 & \begin{array}{cc} 0.3 & 0.3 \end{array}\\ \begin{array}{c} 0.3\\ 0.3 \end{array} & \begin{array}{cc} \begin{array}{c} 1\\ 0.3 \end{array} & \begin{array}{c} 0.3\\ 1 \end{array} \end{array} \end{array} \end{array}\right].$$

Exposure X is generated as binomial distribution $B\left(p_{z}\right)$ with $p_{z}\left(X=1|Z_{1},\cdots ,Z_{10}\right)=\frac{1}{1+e^{-\left(l_{0}+l_{1}Z_{1}+\cdot +l_{10}Z_{10}\right)}}$ , where $l={\left(l_{1},\cdots ,l_{10}\right)}^{T}={\left(0.1,0.3,0.4,0.4,0.6,0.1,0.3,0.4,0.4,0.6\right)}^{T}$ ;

Mediators M and outcome Y depend mainly on the settings of $\Theta_{k}$ , Φ , and the mediation effect αβ . Let $\Theta_{k}={\left(\theta_{k1},\cdots ,\theta_{k10}\right)}^{T}={\left(0.2,0.3,0.3,0.5,0.6,0.2,0.3,0.3,0.5,0.6\right)}^{T}$ be the effect of Z on each $M_{k}$ . For simplicity, we set all $\Theta_{k}$ the same. Let $\Phi ={\left(\phi_{1},\cdots ,\phi_{10}\right)}^{T}={\left(0.1,0.3,0.4,0.4,0.6,0.1,0.3,0.4,0.4,0.6\right)}^{T}$ be the effect of Z on Y . In addition, the terms $c,\gamma ,c_{k},\xi_{k},e_{k}$ are generated by the following patterns:

$$c_{k}∼\;U\left(0,2\right);\;e_{k}∼\;N\left(0,1.2\right);\;\xi_{k}∼\;N\left(0,1\right);\;c=0.5,\gamma =0.5.$$

In order to cover most scenarios in practical application, two sample size levels ( n=300 and n=500 ) and two dimension levels ( p=1000 and p=10000 ) are explored with three mediation effect generation modes as shown below:

(1) Mode 1: Let α=0.6t and β=0.4t for the first eight elements, where t=(0.50,0.60,0.75,0.80,1.00,1.20,1.50,2.00) ; the following four elements ( $\alpha_{9},\alpha_{10},\alpha_{11},\alpha_{12})=(0,0,1.20,1.20)$ , ( $\beta_{9},\beta_{10},\beta_{11},\beta_{12})=(0.80,0.80,0,0)$ ; the other elements are all 0. That is:

$$\alpha ={\left(\underset{\alpha_{1},\ldots ,\alpha_{12}}{\underset{︸}{0.30,0.36,0.45,0.48,0.60,0.72,0.90,1.20,0,0,1.20,1.20}},\underset{\alpha_{13},\ldots ,\alpha_{p}}{\underset{︸}{0,\ldots ,0}}\right)}^{T},$$

$$\beta ={\left(\underset{\beta_{1},\ldots ,\beta_{12}}{\underset{︸}{0.20,0.24,0.30,0.36,0.40,0.48,0.60,0.80,0.80,0.80,0,0}},\underset{\beta_{13},\ldots ,\beta_{p}}{\underset{︸}{0,\ldots ,0}}\right)}^{T}.$$

(2) Mode 2: Let α=0.5t and β=0.5t for the first eight elements, and the other settings are similar with those of mode 1:

$$\alpha ={\left(\underset{\alpha_{1},\ldots ,\alpha_{12}}{\underset{︸}{0.25,0.30,0.375,0.40,0.50,0.60,0.75,1.00,0,0,1.00,1.00}},\underset{\alpha_{13},\ldots ,\alpha_{p}}{\underset{︸}{0,\ldots ,0}}\right)}^{T},$$

$$\beta ={\left(\underset{\beta_{1},\ldots ,\beta_{12}}{\underset{︸}{0.25,0.30,0.375,0.40,0.50,0.60,0.75,1.00,1.00,1.00,0,0}},\underset{\beta_{13},\ldots ,\beta_{p}}{\underset{︸}{0,\ldots ,0}}\right)}^{T}.$$

(3) Mode 3: Let α=0.4t and β=0.6t for the first 8 elements, and the other settings are similar with those of mode 1:

$$\alpha ={\left(\underset{\alpha_{1},\ldots ,\alpha_{12}}{\underset{︸}{0.20,0.24,0.30,0.36,0.40,0.48,0.60,0.80,0,0,0.80,0.80}},\underset{\alpha_{13},\ldots ,\alpha_{p}}{\underset{︸}{0,\ldots ,0}}\right)}^{T},$$

$$\beta ={\left(\underset{\beta_{1},\ldots ,\beta_{12}}{\underset{︸}{0.30,0.36,0.45,0.48,0.60,0.72,0.90,1.20,1.20,1.20,0,0}},\underset{\beta_{13},\ldots ,\beta_{p}}{\underset{︸}{0,\ldots ,0}}\right)}^{T}.$$

It should be noted that in our settings, only the first eight mediators are non-zero, which satisfy the condition $\alpha_{k}\beta_{k}\neq 0$ . Each simulation was repeated 500 times with the seeds 1–500. In addition to the proposed models, we conducted the regression adjustment model that directly includes all confounders Z as covariates into the mediation analysis procedure for comparison.

**FIGURE 3:**
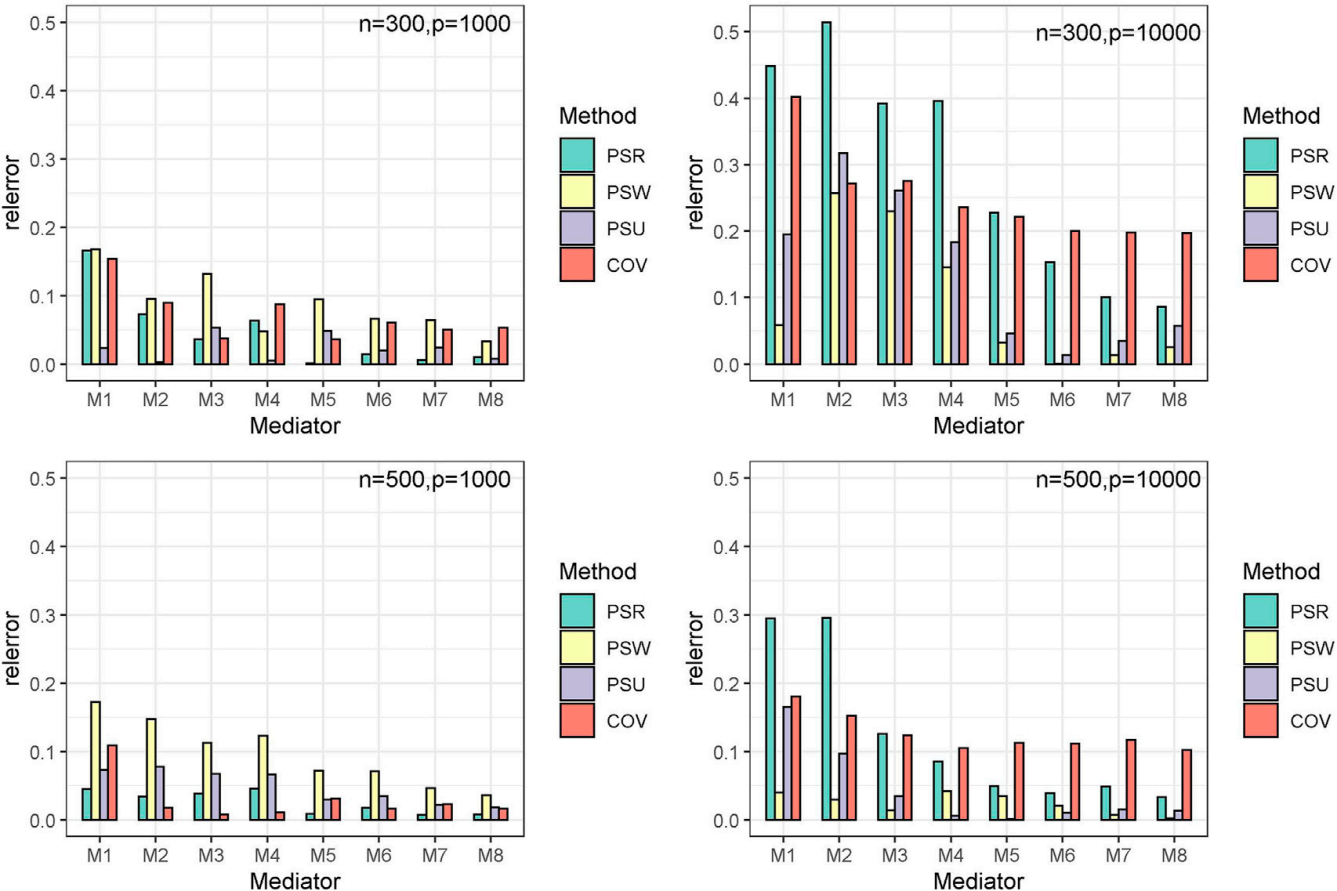
The relative estimate error for the true mediators ( α=0.6t, β=0.4t ). * Relative estimate error=|estimate value – true value| / true value

The simulation results are similar among the three modes. Only results of mode 1 are shown in Tables 1–3 and Figure 3. The other results are provided in Supplementary Material S1.

**TABLE 1:** Correct selection numbers for the eight true mediators (M1–M8) (α=0.6t, β=0.4t )

	Methods	MCP correct selection numbers							
		M1 (αβ = 0.0600)	M2 (αβ = 0.0864)	M3 (αβ = 0.1350)	M4 (αβ = 0.1536)	M5 (αβ = 0.2400)	M6 (αβ = 0.3456)	M7 (αβ = 0.5400)	M8 (αβ = 0.9600)
N = 300 p = 1,000	PSR	245	315	385	383	448	486	499	500
	PSW	305	372	438	454	491	500	500	500
	PSU	305	372	438	454	491	500	500	500
	COV	298	379	457	461	493	499	500	500
N = 300 p = 10,000	PSR	75	111	173	197	328	390	474	500
	PSW	104	163	257	301	431	477	499	500
	PSU	104	163	257	301	431	477	499	500
	COV	110	199	302	340	452	492	500	500
N = 500 p = 1,000	PSR	354	441	472	485	494	500	500	500
	PSW	422	467	489	498	499	500	500	500
	PSU	422	467	489	498	499	500	500	500
	COV	424	467	496	496	500	500	500	500
N = 500 p = 10,000	PSR	163	234	351	391	476	497	499	500
	PSW	213	319	424	455	492	499	500	500
	PSU	213	319	424	455	492	499	500	500
	COV	256	363	440	476	499	500	500	500

* Correct selection numbers measure the total selection number by MCP-penalized regression for each mediator (out of 500 simulation repeats).

Tables 1 describes the mediator correct selection numbers by MCP out of 500 repeats, and Table 2 describes the testing performance by measuring the truth positive rate (TPR), the false positive rate (FP), and the false discovery rate (FDR). Under most settings, both the correct select numbers and TPR are ranked consistently as COV > PSU > PSR > PSW, and the FP is ranked as PSW > PSU > COV > PSR. For example, when detecting the mediator $M_{4}$ with a sample size n=300 and p=10000 , the TPR is 0.448 for COV, 0.388 for PSU, 0.330 for PSW, and 0.240 for PSR. As for the average FP, the value is 0.436 for PSW, 0.256 for PSU, 0.136 for COV, and 0.088 for PSR (sample size n=300 and p=10000 ). All models keep FP at a very low level, with an average value of less than 0.5 per test, which can be negligible. In addition, the PSU model has the most sufficient control of FDR, of which the value is the closest to (and does not exceed) the type I error rate of 0.05. Take the case with a sample size n=300 and p=10000 as an illustration. The FDR for PSU is 0.0447, 0.0173 for PSR, 0.0706 for PSW, and 0.0229 for COV. Notably, the PSU model is the least conservative one among the four models.

**TABLE 2:** TPR, FP, and FDR for the eight true mediators (M1–M8) (α=0.6t, β=0.4t ).

	Methods	TPR								FP	FDR
		M1 (αβ = 0.0600)	M2 (αβ = 0.0864)	M3 (αβ = 0.1350)	M4 (αβ = 0.1536)	M5 (αβ = 0.2400)	M6 (αβ = 0.3456)	M7 (αβ = 0.5400)	M8 (αβ = 0.9600)		
N = 300 p = 1,000	PSR	0.086	0.252	0.442	0.476	0.772	0.928	0.992	1	0.106	0.0161
	PSW	0.178	0.29	0.462	0.51	0.746	0.84	0.954	0.994	0.344	0.0515
	PSU	0.16	0.3	0.514	0.588	0.862	0.962	0.996	1	0.194	0.0302
	COV	0.182	0.368	0.61	0.654	0.896	0.978	1	1	0.142	0.0212
N = 300 p = 10,000	PSR	0.03	0.066	0.182	0.24	0.536	0.744	0.942	1	0.088	0.0173
	PSW	0.044	0.126	0.244	0.33	0.624	0.83	0.952	0.99	0.436	0.0706
	PSU	0.052	0.126	0.296	0.388	0.73	0.924	0.994	1	0.256	0.0447
	COV	0.056	0.174	0.364	0.448	0.78	0.966	0.996	1	0.136	0.0229
N = 500 p = 1,000	PSR	0.362	0.608	0.828	0.892	0.976	1	1	1	0.178	0.0223
	PSW	0.376	0.55	0.714	0.796	0.902	0.966	0.994	1	0.436	0.0521
	PSU	0.42	0.65	0.85	0.91	0.99	1	1	1	0.248	0.0302
	COV	0.488	0.706	0.912	0.926	0.994	1	1	1	0.156	0.0186
N = 500 p = 10,000	PSR	0.158	0.286	0.604	0.708	0.938	0.994	0.998	1	0.162	0.0239
	PSW	0.184	0.362	0.596	0.706	0.886	0.97	0.994	0.998	0.432	0.0559
	PSU	0.218	0.418	0.74	0.824	0.972	0.998	1	1	0.262	0.0354
	COV	0.244	0.442	0.764	0.872	0.988	1	1	1	0.166	0.0221

*TPR measures the true positive rate towards each true mediator (M1–M8); FP is the false positive number; and FDR is the false discovery rate (= FP/TP, where TP is the total positive numbers). All the indicators are the average over the 500 simulation repeats.

Table 3 presents the estimate and mean square error (MSE) for the indirect effects $\alpha_{k}\beta_{k}$ ; Figure 1 shows the relative estimate error histogram. The estimators approach the true value as the indirect effect increases ( $M_{1}-M_{8}$ ), and all models tend to be accurate when n gets larger and p gets smaller. Among the four models, the PSU model shows the most stability, with the lowest relative error in most cases. For example, when sample size n = 300 and dimension *p* = 1000, the max relative estimate error of the PSU model is around 0.05, while the other three models are all close to 0.15. As for the performance of other models, PSR is sensitive to the conditions and works best when the data information is sufficient ( n=500,p=1000 ), while the PSW model behaves inversely. The traditional COV model shows the biggest bias, and results show that under insufficient sample conditions (sample size n=300,p=10000 ), the relative estimate error of the COV model toward $M_{4}-M_{7}$ is almost invariant around 0.15–0.20, indicating there is a fixed bias when adjusting for confounders by the COV model.

**TABLE 3:** Estimation results of mediation effects (αβ) ( α=0.6t, β=0.4t ).

(*α* = 0.6t, *β* = 0.4t) (MSE)		(0.30,0.20) = 0.0600 (MSE)	(0.36,0.24) = 0.0864 (MSE)	(0.45,0.30) = 0.1350 (MSE)	(0.48,0.32) = 0.1536 (MSE)	(0.60,0.40) = 0.2400 (MSE)	(0.72,0.48) = 0.3456 (MSE)	(0.90,0.60) = 0.5400 (MSE)	(1.20,0.80) = 0.9600 (MSE)
*N* = 300 p = 1,000	PSR	0.0500 (0.0036)	0.0800 (0.0031)	0.1302 (0.0054)	0.1438 (0.0052)	0.2397 (0.0078)	0.3405 (0.0090)	0.5431 (0.0133)	0.9503 (0.0207)
	PSW	0.0701 (0.0055)	0.0946 (0.0042)	0.1528 (0.0075)	0.1609 (0.0072)	0.2627 (0.0100)	0.3685 (0.0146)	0.5748 (0.0255)	0.9915 (0.0423)
	PSU	0.0586 (0.0037)	0.0862 (0.0027)	0.1422 (0.0044)	0.1528 (0.0046)	0.2516 (0.0054)	0.3525 (0.0083)	0.5531 (0.0139)	0.9677 (0.0209)
	COV	0.0508 (0.0031)	0.0787 (0.0023)	0.1299 (0.0033)	0.1402 (0.0035)	0.2313 (0.0047)	0.3247 (0.0069)	0.5128 (0.0121)	0.9092 (0.0195)
*N* = 300 p = 10,000	PSR	0.0331 (0.0063)	0.0420 (0.0035)	0.0821 (0.0064)	0.0928 (0.0076)	0.1854 (0.0121)	0.2928 (0.0151)	0.4858 (0.0184)	0.8777 (0.0219)
	PSW	0.0635 (0.0089)	0.0642 (0.0065)	0.1040 (0.0094)	0.1313 (0.0103)	0.2322 (0.0122)	0.3454 (0.0144)	0.5326 (0.0214)	0.9356 (0.0393)
	PSU	0.0483 (0.0058)	0.0590 (0.0043)	0.0998 (0.0066)	0.1255 (0.0073)	0.2289 (0.0086)	0.3409 (0.0092)	0.5214 (0.0137)	0.9044 (0.0235)
	COV	0.0359 (0.0034)	0.0629 (0.0025)	0.0978 (0.0038)	0.1174 (0.0033)	0.1868 (0.0047)	0.2764 (0.0058)	0.4331 (0.0100)	0.7711 (0.0199)
**(α = 0.6t, β = 0.4t) (MSE)**		**(0.30,0.20) = 0.0600 (MSE)**	**(0.36,0.24) = 0.0864< (MSE)**	**(0.45,0.30) = 0.1350 (MSE)**	**(0.48,0.32) = 0.1536 (MSE)**	**(0.60,0.40) = 0.2400 (MSE)**	**(0.72,0.48) = 0.3456 (MSE)**	**(0.90,0.60) = 0.5400 (MSE)**	**(1.20,0.80) = 0.9600 (MSE)**
*N* = 500 p = 1,000	PSR	0.0573 (0.0021)	0.0893 (0.0016)	0.1402 (0.0023)	0.1606 (0.0022)	0.2420 (0.0032)	0.3516 (0.0043)	0.5438 (0.0079)	0.9678 (0.0107)
	PSW	0.0703 (0.0020)	0.0991 (0.0023)	0.1501 (0.0040)	0.1724 (0.0037)	0.2572 (0.0054)	0.3701 (0.0077)	0.5649 (0.0122)	0.9945 (0.0207)
	PSU	0.0644 (0.0012)	0.0931 (0.0014)	0.1441 (0.0023)	0.1638 (0.0022)	0.2470 (0.0031)	0.3575 (0.0044)	0.5517 (0.0077)	0.9773 (0.0122)
	COV	0.0535 (0.0016)	0.0849 (0.0012)	0.1339 (0.0017)	0.1519 (0.0020)	0.2325 (0.0028)	0.3399 (0.0039)	0.5277 (0.0071)	0.9447 (0.0119)
*N* = 500 p = 10,000	PSR	0.0423 (0.0032)	0.0609 (0.0025)	0.1180 (0.0038)	0.1405 (0.0037)	0.2282 (0.0040)	0.3320 (0.0047)	0.5138 (0.0070)	0.9279 (0.0116)
	PSW	0.0576 (0.0039)	0.0839 (0.0030)	0.1369 (0.0046)	0.1601 (0.0043)	0.2484 (0.0055)	0.3527 (0.0073)	0.5438 (0.0120)	0.9617 (0.0200)
	PSU	0.0501 (0.0026)	0.0780 (0.0023)	0.1303 (0.0031)	0.1545 (0.0029)	0.2398 (0.0034)	0.3421 (0.0045)	0.5316 (0.0077)	0.9478 (0.0122)
	COV	0.0492 (0.0011)	0.0733 (0.0015)	0.1183 (0.0021)	0.1375 (0.0020)	0.2130 (0.0026)	0.3071 (0.0038)	0.4770 (0.0063)	0.8622 (0.0123)

a The estimation value of mediation effect (or MSE) for each mediator is calculated as the average (or standard error) of the corresponding mediators that are selected by MCP over the 500 simulation repeats; PSR, the propensity score regression method; PSW, the propensity score weighting method; PSU, the hybrid method; COV, the traditional covariate regression method.

Overall, although the COV model has the highest TPR, it shows a large bias when estimating the mediation effects. The PSU model is the most recommended, which performs best in estimating and is only second to the COV model in testing.

## 4 Data application

Smoking is a major environmental hazard promoting lung disease development. Previous studies have demonstrated that smoking can lead to some abnormal expression of CpG islands (DNA methylation sites) in lung-related genes, which may be the immediate cause of lung disease (Toyooka et al., 2003; Harlid et al., 2014). Generally, DNA methylation data can be obtained by the technology Infinium HumanMethylation450, resulting in a dataset of more than 480,000 CpG sites over the whole genome (Dedeurwaerder et al., 2011). Hence, we conducted high-dimensional mediation analysis to further discover the specific functional CpG sites that mediate the relationship between smoking and lung disease.

Clinical and methylation data from the cohorts of lung squamous cell carcinoma (LUSC) and lung adenocarcinoma (LUAD) were used for analysis. The clinical datasets included 626 and 706 samples, respectively, and the methylation dataset included 485,577 probes. Baseline information such as age, sex, and race were collected, and DLCO (diffusing capacity of the lung for carbon monoxide) was measured to characterize the lung function of every individual. Subjects were categorized into the non-smoker group and smoker group according to their smoking status.

After removing the subjects with “not available,” there were 254 samples in the smoker group and 119 samples in the non-smoker group. As shown in Table 4, the baseline variables such as age, race, gender, and the outcome variable DLCO show marginally significant differences between the smoking groups, indicating the necessity to adjust for confounders in the following analysis.

**TABLE 4:** Clinical characteristics of the patients in the smoker (S) and non-smoker (NS) groups.

Variable		Total (n = 373)	Smoker (*n* = 254)	Non-smoker (*n* = 119)	*p*-value
Age (std)		66.32 (10.09)	64.27 (9.69)	70.69 (9.55)	6.57 × 10^(−09)^
Gender	Male	217	156	61	0.082
	Female	156	98	58	
Race	White	269	173	96	0.040
	Others	104	81	23	
DLCO(std)		70.52 (21.63)	67.62 (22.16)	76.70 (19.12)	6.53 × 10^(−05)^

Table 5 summarizes the analysis results. We focused on methylation sites with a %TE (total effect proportion) greater than 10. Cg24480765 in the gene RP11-347H15.2 was a significant mediation site detected by all models, whose mediation effect αβ is around 0.11 × 0.82. The results reveal that smoking will promote the demethylation of Cg24480765, leading to an increase in gene expression and ultimately reducing the DLCO level. In other words, the gene RP11-347H15.2 that Cg24480765 locates may be a proto-oncogene. We have not found direct research on gene RP11-347H15.2. However, the gene belongs to the LncRNA family and much literature has stated that LncRNA can be an important molecular marker of various cancers and is closely related to the occurrence of cancer (Huarte, 2015; Schmitt and Chang, 2016). Thus, future insights into the gene RP11-357H15.2 will be meaningful.

**TABLE 5:** Summary of the selected CpGs by the proposed models.

Method	CpG	Gene	Chrom	$\hat{\alpha }$	$\hat{\beta }$	%TE	*p*-value (FDR)
**PSR**	cg24480765	RP11-347H15.2	chr11	−0.1099	0.8225	18.0072	0.0022
	cg13835688	SLC25A25	chr9	0.0207	−1.9684	8.1322	0.0103
**PSW**	cg24480765	RP11-347H15.2	chr11	−0.1079	0.8225	16.62	0.0003
	cg22051776	KLF3	chr4	0.0291	−2.0930	11.38	0.0203
	cg22664428	DGCR11, DGCR2	chr22	0.0305	1.5568	−8.89	0.0041
	cg08763422	WHSC1	chr4	0.0498	−0.5382	5.02	0.0125
**PSU**	cg24480765	RP11-347H15.2	chr11	−0.1099	0.8225	16.9259	0.0013
	cg22051776	KLF3	chr4	0.0328	−2.0930	12.8584	0.0203
	cg22664428	DGCR11, DGCR2	chr22	0.0325	1.5568	−9.4776	0.0041
	cg08763422	WHSC1	chr4	0.0497	−0.5382	5.0019	0.0456

We identified another site, Cg22051776, in the KLF3 gene by models PSU and PSW. The indirect mediation effect αβ is about +0.03×−2.5 , suggesting that smoking will promote the DNA methylation of the site to repress the gene expression and finally reduce the DLCO level. The causal chain means that the KLF3 gene may inhibit lung disease. Similarly, existing studies have proved that KLF3 is an important tumor suppressor gene of lung adenocarcinoma, and KLF3 silencing promotes the EMT process in lung cancer (Zhu et al., 2012; Sun et al., 2019). The consistency of experimental literature and our data-driven inference verifies the accuracy and reliability of our models to some extent.

## 5 Discussion

The unbiased high-dimensional mediation inference needs to satisfy the no-confounding assumption. However, confounding is almost inevitable in observational HIMA cases because of the non-randomization of the baseline covariates. To solve the problem, we adopted the HIMA framework of SIS, MCP, and joint-significance testing, and combined it with three propensity score utilization methods to adjust for confounders. We compared them to the regression adjustment method (COV model) that takes all confounders as covariates. Simulation results show that our proposed model PSU performs the best from the overall perspective of estimation accuracy, TPR, FP, FDR, and model simplicity. Finally, we applied our models to the TCGA lung cancer dataset and found the important DNA methylation mediators, cg24480765 and cg22051776. Particularly, our utilization of propensity scores is not just limited to HIMA. It gives an idea of adjusting for confounders under other causal inference cases.

Still, there are some improvements worth discussion in the future. First, the HIMA framework we adopted can be developed in some aspects. For example, Gao et al. (2019) used a de-biased lasso estimator in the variable selection part, and developed a new model called HDMA, which can deal with the correlation between methylation sites better. In addition, applying other weighting methods such as the stable weights proposed by Zubizarreta (2015) in the PSW and PSU models might help to enhance the model robustness. As for the significance testing part, the joint-significance testing we used may be conservative (Dai et al., 2022) and other testing methods such as bootstrapping would be more powerful. MacKinnon et al. revealed that the bias-corrected bootstrap is the best method for testing indirect effects (MacKinnon et al., 2004); Benjamini and Yekutieli. (2005) introduced a procedure of getting FDR-adjusted multiple confidence intervals for selected parameters. Yet our research did not focus much on the testing part. Moreover, the exposure variable in our model is set to be binary. Continuous variables or discrete variables with more than two groups need further expansion.

## Data Availability

Our numerical analysis is implemented by R and the corresponding code is available in https://github.com/linghaoluo/PS_HIMA_CON.
