# Supplementary material for: Linear high-dimensional mediation models adjusting for confounders using propensity score method
Source: Front Genet. 2022 Oct 10;13:961148. doi: 10.3389/fgene.2022.961148 (PMC9589256; doi:10.3389/fgene.2022.961148)
Supplement: Supplementary file 1 [file DataSheet1.docx]

Supplementary Material

# Supplementary Figures and Tables

## Supplementary Figures


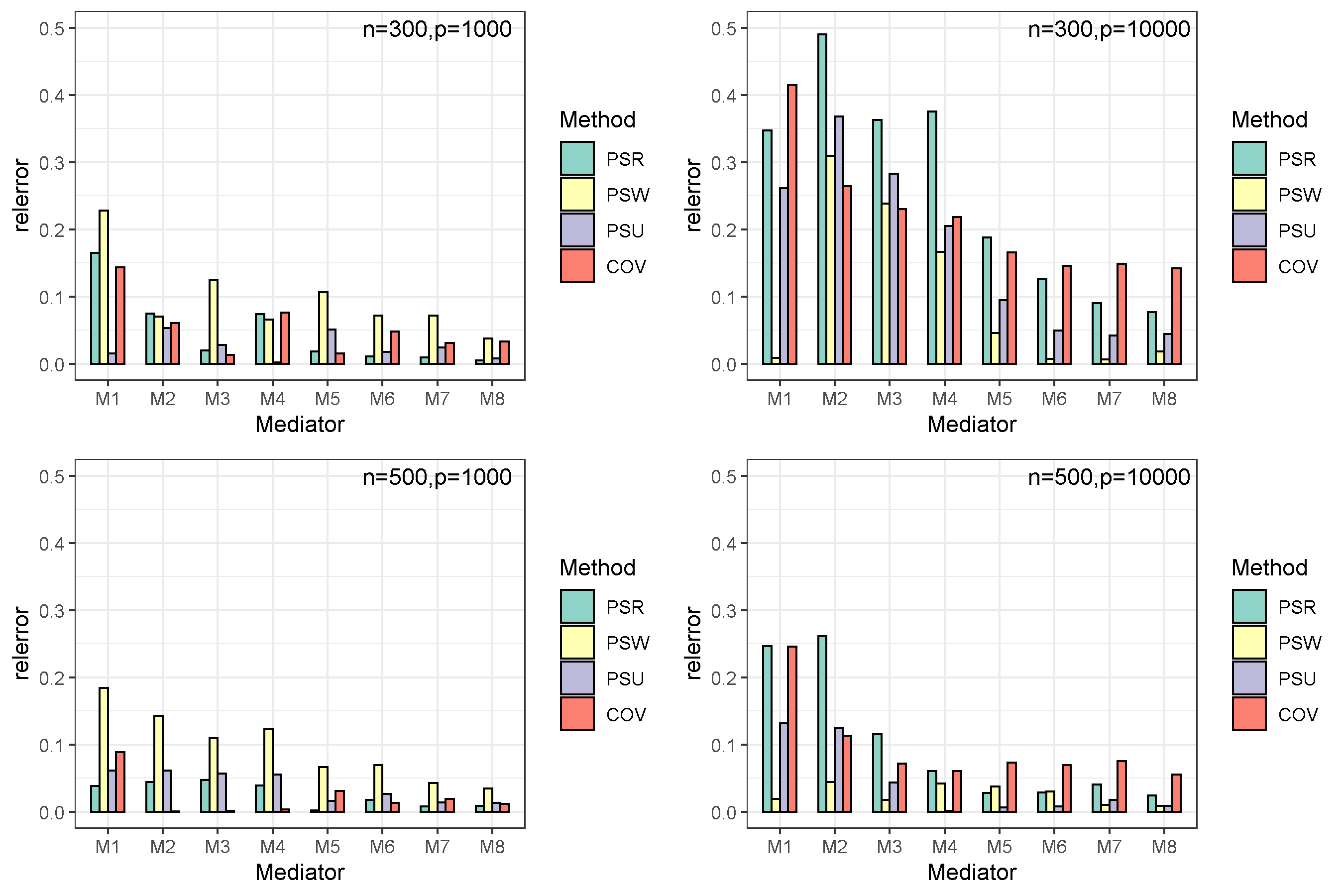


**Supplementary Figure 1.** The relative estimate error for the true mediators ($\alpha=0.5t, \beta=0.5t$).

*$Relative estimate error = |estimate value - true value| / true value$


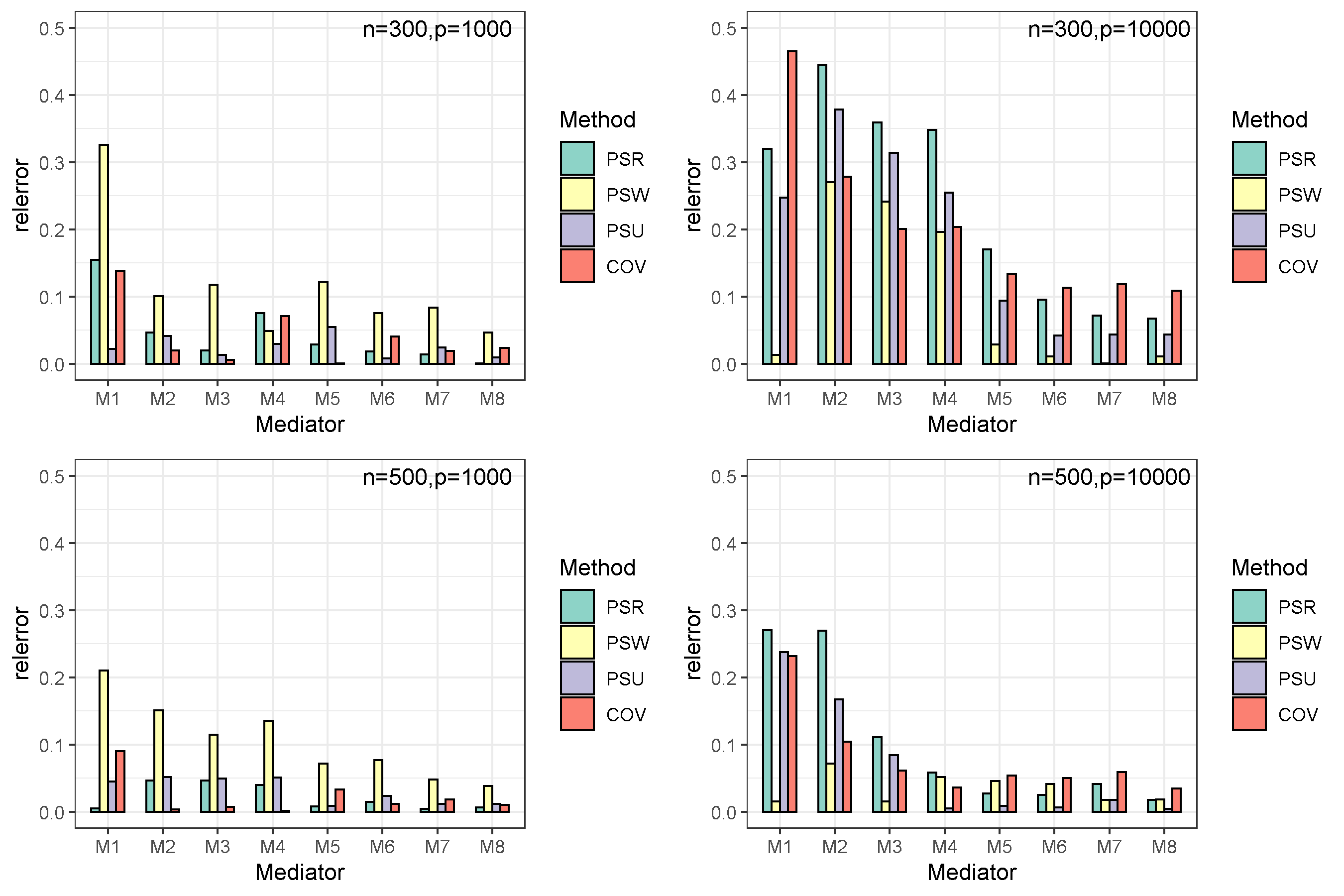


**Supplementary Figure 2.** The relative estimate error for the true mediators ($\alpha=0.4t, \beta=0.6t$). *$Relative estimate error = |estimate value - true value| / true value$

## Supplementary Tables

**Supplementary Table 1 |** Estimation results of mediation effects $\left( \alpha\beta\right)$ ($\alpha=0.5t, \beta=0.5t$)

| **(α=0.5t, β=0.5t) (MSE)** | | **(0.25,0.25)=0.0625 (MSE)** | **(0.30,0.30)=0.0900 (MSE)** | **(0.375,0.375)=0.1406 (MSE)** | **(0.40,0.40)=0.1600 (MSE)** | **(0.50,0.50)=0.2500 (MSE)** | **(0.60,0.60)=0.3600 (MSE)** | **(0.75,0.75)=0.5625 SE)** | **(1.00,1.00)=1.0000 (MSE)** |
| --- | --- | --- | --- | --- | --- | --- | --- | --- | --- |
| **N=300**  **P=1000** | **PSR** | 0.0522 (0.0059) | 0.0833 (0.0037) | 0.1378 (0.0063) | 0.1482 (0.0064) | 0.2546 (0.0089) | 0.3560 (0.0120) | 0.5677 (0.0182) | 0.9953 (0.0281) |
|  | **PSW** | 0.0768 (0.0078) | 0.0963 (0.0058) | 0.1581 (0.0107) | 0.1705 (0.0097) | 0.2767 (0.0150) | 0.3857 (0.0208) | 0.6030 (0.0363) | 1.0373 (0.0617) |
|  | **PSU** | 0.0635 (0.0049) | 0.0853 (0.0037) | 0.1445 (0.0061) | 0.1597 (0.0059) | 0.2627 (0.0081) | 0.3663 (0.0113) | 0.5762 (0.0188) | 1.0076 (0.0284) |
|  | **COV** | 0.0535 (0.0042) | 0.0846 (0.0028) | 0.1387 (0.0041) | 0.1478 (0.0049) | 0.2462 (0.0067) | 0.3427 (0.0100) | 0.5450 (0.0166) | 0.9666 (0.0276) |
| **N=300 P=10000** | **PSR** | 0.0408 (0.0097) | 0.0459 (0.0041) | 0.0896 (0.0069) | 0.0999 (0.0087) | 0.2031 (0.0133) | 0.3147 (0.0170) | 0.5119 (0.0220) | 0.9229 (0.0300) |
|  | **PSW** | 0.0630 (0.0176) | 0.0621 (0.0089) | 0.1070 (0.0122) | 0.1333 (0.0134) | 0.2386 (0.0174) | 0.3574 (0.0226) | 0.5588 (0.0310) | 0.9820 (0.0560) |
|  | **PSU** | 0.0462 (0.0094) | 0.0569 (0.0052) | 0.1008 (0.0080) | 0.1272 (0.0088) | 0.2263 (0.0118) | 0.3422 (0.0141) | 0.5391 (0.0019) | 0.9561 (0.0311) |
|  | **COV** | 0.0366 (0.0064) | 0.0662 (0.0036) | 0.1082 (0.0048) | 0.1250 (0.0046) | 0.2086 (0.0063) | 0.3076 (0.0087) | 0.4788 (0.0142) | 0.8580 (0.0275) |
| **(α=0.5t, β=0.5t) (MSE)** | | **(0.25,0.25)=0.0625 (MSE)** | **(0.30,0.30)=0.0900 (MSE)** | **(0.375,0.375)=0.1406 (MSE)** | **(0.40,0.40)=0.1600 (MSE)** | **(0.50,0.50)=0.2500 (MSE)** | **(0.60,0.60)=0.3600 (MSE)** | **(0.75,0.75)=0.5625 SE)** | **(1.00,1.00)=1.0000 (MSE)** |
| **N=500**  **P=1000** | **PSR** | 0.0601 (0.0026) | 0.0940 (0.0020) | 0.1472 (0.0028) | 0.1663 (0.0031) | 0.2504 (0.0045) | 0.3662 (0.0060) | 0.5668 (0.0109) | 1.0084 (0.0173) |
|  | **PSW** | 0.0740 (0.0028) | 0.1028 (0.0032) | 0.1560 (0.0055) | 0.1796 (0.0052) | 0.2665 (0.0076) | 0.3849 (0.0110) | 0.5865 (0.0175) | 1.0344 (0.0299) |
|  | **PSU** | 0.06633 (0.0016) | 0.0955 (0.0019) | 0.1486 (0.0030) | 0.1689 (0.0030) | 0.2540 (0.0042) | 0.3694 (0.0060) | 0.5702 (0.0108) | 1.0131 (0.0172) |
|  | **COV** | 0.0569 (0.0020) | 0.0900 (0.0015) | 0.1404 (0.0023) | 0.1595 (0.0026) | 0.2423 (0.0039) | 0.3553 (0.0054) | 0.5520 (0.0099) | 0.9883 (0.0164) |
| **N=500**  **P=10000** | **PSR** | 0.0471 (0.0035) | 0.0665 (0.0029) | 0.1244 (0.0045) | 0.1503 (0.0044) | 0.2431 (0.0045) | 0.3497 (0.0061) | 0.5396 (0.0097) | 0.9758 (0.0159) |
|  | **PSW** | 0.0637 (0.0056) | 0.0861 (0.0040) | 0.1431 (0.0064) | 0.1667 (0.0064) | 0.2594 (0.0083) | 0.3707 (0.0108) | 0.5683 (0.0175) | 1.0090 (0.0291) |
|  | **PSU** | 0.0543 (0.0035) | 0.0788 (0.0031) | 0.1345 (0.0041) | 0.1598 (0.0042) | 0.2484 (0.0051) | 0.3572 (0.0063) | 0.5527 (0.0105) | 0.9912 (0.0164) |
|  | **COV** | 0.0471 (0.0024) | 0.0799 (0.0019) | 0.1305 (0.0026) | 0.1503 (0.0029) | 0.2317 (0.0038) | 0.3350 (0.0052) | 0.5202 (0.0092) | 0.9443 (0.0161) |

* The estimation value of mediation effect (or MSE) for each mediator is calculated as the average (or standard error) of the corresponding mediators that are selected by MCP over the 500 simulation repeats; PSR, the propensity score regression method; PSW, the propensity score weighting method; PSU, the hybrid method; COV, the traditional covariate regression method.

**Supplementary Table 2 |** Estimation results of mediation effects $\left( \alpha\beta\right)$($\alpha=0.4t, \beta=0.6t$)

| **(α=0.4t, β=0.6t) (MSE)** | | **(0.20,0.30)=0.0600 (MSE)** | **(0.24,0.36)=0.0864 (MSE)** | **(0.30,0.45)=0.1350 (MSE)** | **(0.32,0.48)=0.1536 (MSE)** | **(0.40,0.60)=0.2400 (MSE)** | **(0.48,0.72)=0.3456 (MSE)** | **(0.60,0.80)=0.5400 (MSE)** | **(0.80,1.20)=0.9600 (MSE)** |
| --- | --- | --- | --- | --- | --- | --- | --- | --- | --- |
| **N=300**  **P=1000** | **PSR** | 0.0507 (0.0069) | 0.0824 (0.0043) | 0.1323 (0.0077) | 0.1420 (0.0077) | 0.2469 (0.0109) | 0.3394 (0.0157) | 0.5474 (0.0247) | 0.9597 (0.0382) |
|  | **PSW** | 0.0795 (0.0104) | 0.0951 (0.0076) | 0.1509 (0.0147) | 0.1611 (0.0133) | 0.2693 (0.0205) | 0.3715 (0.0299) | 0.5851 (0.0508) | 1.0043 (0.0852) |
|  | **PSU** | 0.0613 (0.0068) | 0.0828 (0.0043) | 0.1367 (0.0079) | 0.1491 (0.0077) | 0.2531 (0.0106) | 0.3483 (0.0162) | 0.5531 (0.0260) | 0.9689 (0.0379) |
|  | **COV** | 0.0517 (0.0058) | 0.0847 (0.0034) | 0.1342 (0.0056) | 0.1427 (0.0062) | 0.2402 (0.0090) | 0.3317 (0.0137) | 0.5298 (0.0225) | 0.9373 (0.0372) |
| **N=300 P=10000** | **PSR** | 0.0408 (0.0140) | 0.0480 (0.0044) | 0.0865 (0.0081) | 0.1001 (0.0097) | 0.1992 (0.0149) | 0.3127 (0.0185) | 0.5013 (0.0254) | 0.8958 (0.0380) |
|  | **PSW** | 0.0592 (0.0266) | 0.0630 (0.0081) | 0.1024 (0.0143) | 0.1235 (0.0158) | 0.2331 (0.0219) | 0.3494 (0.0294) | 0.5401 (0.0417) | 0.9499 (0.0753) |
|  | **PSU** | 0.0452 (0.0126) | 0.0537 (0.0050) | 0.0926 (0.0087) | 0.1145 (0.0100) | 0.2174 (0.0136) | 0.3312 (0.0175) | 0.5164 (0.0245) | 0.9183 (0.0393) |
|  | **COV** | 0.0321 (0.0115) | 0.0624 (0.0039) | 0.1079 (0.0060) | 0.1223 (0.0058) | 0.2079 (0.0089) | 0.3066 (0.0118) | 0.4761 (0.0191) | 0.8557 (0.0378) |
| **(α=0.4t, β=0.6t) (MSE)** | | **(0.20,0.30)=0.0600 (MSE)** | **(0.24,0.36)=0.0864 (MSE)** | **(0.30,0.45)=0.1350 (MSE)** | **(0.32,0.48)=0.1536 (MSE)** | **(0.40,0.60)=0.2400 (MSE)** | **(0.48,0.72)=0.3456 (MSE)** | **(0.60,0.80)=0.5400 (MSE)** | **(0.80,1.20)=0.9600 (MSE)** |
| **N=500**  **P=1000** | **PSR** | 0.0597 (0.0028) | 0.0904 (0.0025) | 0.1413 (0.0037) | 0.1597 (0.0040) | 0.2381 (0.0062) | 0.3506 (0.0080) | 0.5422 (0.0146) | 0.9661 (0.0229) |
|  | **PSW** | 0.0726 (0.0038) | 0.0995 (0.0045) | 0.1504 (0.0075) | 0.1744 (0.0071) | 0.2572 (0.0107) | 0.3722 (0.0152) | 0.5657 (0.0244) | 0.9968 (0.0413) |
|  | **PSU** | 0.0627 (0.0028) | 0.0901 (0.0026) | 0.1416 (0.0041) | 0.1614 (0.0041) | 0.2421 (0.0058) | 0.3536 (0.0080) | 0.5461 (0.0148) | 0.9713 (0.0230) |
|  | **COV** | 0.0546 (0.0027) | 0.0861 (0.0020) | 0.1360 (0.0032) | 0.1534 (0.0036) | 0.2321 (0.0053) | 0.3417 (0.0074) | 0.5301 (0.0137) | 0.9506 (0.0222) |
| **N=500**  **P=10000** | **PSR** | 0.0438 (0.0047) | 0.0631 (0.0032) | 0.1200 (0.0051) | 0.1446 (0.0051) | 0.2335 (0.0061) | 0.3371 (0.0080) | 0.5179 (0.0135) | 0.9431 (0.0221) |
|  | **PSW** | 0.0591 (0.0112) | 0.0802 (0.0050) | 0.1330 (0.0084) | 0.1615 (0.0086) | 0.2509 (0.0116) | 0.3598 (0.0148) | 0.5493 (0.0243) | 0.9772 (0.0401) |
|  | **PSU** | 0.0457 (0.0057) | 0.0719 (0.0036) | 0.1236 (0.0055) | 0.1528 (0.0053) | 0.2380 (0.0068) | 0.3434 (0.0083) | 0.5304 (0.0141) | 0.9557 (0.0216) |
|  | **COV** | 0.0461 (0.0036) | 0.0774 (0.0023) | 0.1268 (0.0035) | 0.1481 (0.0037) | 0.2271 (0.0053) | 0.3283 (0.0072) | 0.5082 (0.0122) | 0.9271 (0.0211) |

* The estimation value of mediation effect (or MSE) for each mediator is calculated as the average (or standard error) of the corresponding mediators that are selected by MCP over the 500 simulation repeats; PSR, the propensity score regression method; PSW, the propensity score weighting method; PSU, the hybrid method; Cov, the traditional covariate regression method.

**Supplementary Table 3 |** Correct selection numbers for the eight true mediators (M_1_-M_8_) $(\alpha=0.5t, \beta=0.5t$）

| **Sample & Dimension** | **Methods** | **MCP Correct Selection Numbers** | | | | | | | |
| --- | --- | --- | --- | --- | --- | --- | --- | --- | --- |
|  |  | **M_1_**  **(αβ=0.0625)** | **M_2_**  **(αβ=0.0900)** | **M_3_**  **(αβ=0.1406)** | **M_4_**  **(αβ=0.1600)** | **M5**  **(αβ=0.2500)** | **M6**  **(αβ=0.3600)** | **M7**  **(αβ=0.5625)** | **M8**  **(αβ=1.0000)** |
| **N=300**  **P=1000** | **PSR** | 263 | 333 | 394 | 395 | 456 | 489 | 499 | 500 |
|  | **PSW** | 295 | 354 | 420 | 440 | 484 | 500 | 500 | 500 |
|  | **PSU** | 295 | 354 | 420 | 440 | 484 | 500 | 500 | 500 |
|  | **COV** | 335 | 408 | 465 | 471 | 495 | 499 | 500 | 500 |
| **N=300**  **P=10000** | **PSR** | 80 | 126 | 189 | 211 | 337 | 409 | 477 | 500 |
|  | **PSW** | 91 | 155 | 238 | 283 | 403 | 462 | 498 | 500 |
|  | **PSU** | 91 | 155 | 238 | 283 | 403 | 462 | 498 | 500 |
|  | **COV** | 133 | 226 | 333 | 369 | 464 | 495 | 500 | 500 |
| **N=500**  **P=1000** | **PSR** | 371 | 448 | 478 | 488 | 496 | 500 | 500 | 500 |
|  | **PSW** | 414 | 456 | 488 | 493 | 498 | 500 | 500 | 500 |
|  | **PSU** | 414 | 456 | 488 | 493 | 498 | 500 | 500 | 500 |
|  | **COV** | 437 | 476 | 496 | 499 | 500 | 500 | 500 | 500 |
| **N=500**  **P=10000** | **PSR** | 179 | 253 | 366 | 409 | 481 | 498 | 499 | 500 |
|  | **PSW** | 192 | 292 | 408 | 441 | 486 | 498 | 500 | 500 |
|  | **PSU** | 192 | 292 | 408 | 441 | 486 | 498 | 500 | 500 |
|  | **COV** | 279 | 392 | 460 | 480 | 500 | 500 | 500 | 500 |

*Correct selection numbers measures the total selection number by MCP-penalized regression for each mediator (out of 500 simulation repeats).

**Supplementary Table 4.** Correct selection numbers for the eight true mediators (M_1_-M_8_) $(\alpha=0.4t, \beta=0.6t$）

| **Sample & Dimension** | **Methods** | **MCP Selection Times** | | | | | | | |
| --- | --- | --- | --- | --- | --- | --- | --- | --- | --- |
|  |  | **M_1_**  **(αβ=0.0600)** | **M_2_**  **(αβ=0.0864)** | **M_3_**  **(αβ=0.1350)** | **M_4_**  **(αβ=0.1536)** | **M_5_**  **(αβ=0.2400)** | **M_6_**  **(αβ=0.3456)** | **M_7_**  **(αβ=0.5400)** | **M_8_**  **(αβ=0.9600)** |
| **N=300**  **P=1000** | **PSR** | 272 | 342 | 402 | 403 | 460 | 490 | 499 | 500 |
|  | **PSW** | 274 | 335 | 397 | 420 | 475 | 495 | 499 | 500 |
|  | **PSU** | 274 | 335 | 397 | 420 | 475 | 495 | 499 | 500 |
|  | **COV** | 345 | 419 | 471 | 474 | 496 | 499 | 500 | 500 |
| **N=300**  **P=10000** | **PSR** | 87 | 129 | 198 | 223 | 348 | 423 | 481 | 500 |
|  | **PSW** | 85 | 129 | 216 | 251 | 379 | 441 | 496 | 500 |
|  | **PSU** | 85 | 129 | 216 | 251 | 379 | 441 | 496 | 500 |
|  | **COV** | 138 | 239 | 347 | 382 | 470 | 497 | 500 | 500 |
| **N=500**  **P=1000** | **PSR** | 380 | 451 | 480 | 491 | 497 | 500 | 500 | 500 |
|  | **PSW** | 387 | 446 | 483 | 488 | 498 | 500 | 500 | 500 |
|  | **PSU** | 387 | 446 | 483 | 488 | 498 | 500 | 500 | 500 |
|  | **COV** | 441 | 479 | 498 | 499 | 500 | 500 | 500 | 500 |
| **N=500**  **P=10000** | **PSR** | 188 | 262 | 377 | 419 | 484 | 498 | 499 | 500 |
|  | **PSW** | 167 | 266 | 370 | 411 | 472 | 498 | 500 | 500 |
|  | **PSU** | 167 | 266 | 370 | 411 | 472 | 498 | 500 | 500 |
|  | **COV** | 291 | 406 | 466 | 489 | 500 | 500 | 500 | 500 |

*Correct selection numbers measures the total selection number by MCP-penalized regression for each mediator (out of 500 simulation repeats).

**Supplementary Table 5.** TPR, FP and FDR for the eight true mediators (M1-M8) $(\alpha=0.5t, \beta=0.5t$）

| **Sample & Dimension** | **Methods** | **TPR** | | | | | | | | **FP** | **FDR** |
| --- | --- | --- | --- | --- | --- | --- | --- | --- | --- | --- | --- |
|  |  | **M_1_**  **(αβ=0.0625)** | **M_2_**  **(αβ=0.0900)** | **M_3_**  **(αβ=0.1406)** | **M_4_**  **(αβ=0.1600)** | **M_5_**  **(αβ=0.2500)** | **M_6_**  **(αβ=0.3600)** | **M_7_**  **(αβ=0.5625)** | **M_8_**  **(αβ=1.0000)** |  |  |
| **N=300**  **P=1000** | **PSR** | 0.06 | 0.15 | 0.314 | 0.346 | 0.65 | 0.814 | 0.968 | 1 | 0.106 | 0.0181 |
|  | **PSW** | 0.118 | 0.204 | 0.328 | 0.358 | 0.58 | 0.724 | 0.874 | 0.968 | 0.328 | 0.0516 |
|  | **PSU** | 0.106 | 0.166 | 0.34 | 0.39 | 0.684 | 0.826 | 0.97 | 1 | 0.172 | 0.0304 |
|  | **COV** | 0.132 | 0.28 | 0.448 | 0.488 | 0.772 | 0.902 | 0.982 | 1 | 0.108 | 0.0175 |
| **N=300**  **P=10000** | **PSR** | 0.022 | 0.048 | 0.128 | 0.16 | 0.426 | 0.676 | 0.902 | 0.998 | 0.072 | 0.0158 |
|  | **PSW** | 0.038 | 0.09 | 0.176 | 0.22 | 0.452 | 0.672 | 0.858 | 0.956 | 0.414 | 0.0762 |
|  | **PSU** | 0.03 | 0.088 | 0.18 | 0.246 | 0.534 | 0.78 | 0.948 | 0.998 | 0.252 | 0.0494 |
|  | **COV** | 0.032 | 0.116 | 0.298 | 0.34 | 0.67 | 0.88 | 0.972 | 0.998 | 0.106 | 0.0194 |
| **N=500**  **P=1000** | **PSR** | 0.248 | 0.454 | 0.694 | 0.762 | 0.93 | 0.99 | 0.998 | 1 | 0.16 | 0.0215 |
|  | **PSW** | 0.27 | 0.432 | 0.576 | 0.616 | 0.79 | 0.92 | 0.976 | 0.998 | 0.45 | 0.0603 |
|  | **PSU** | 0.27 | 0.46 | 0.686 | 0.77 | 0.932 | 0.99 | 1 | 1 | 0.276 | 0.0369 |
|  | **COV** | 0.382 | 0.56 | 0.784 | 0.846 | 0.964 | 0.998 | 1 | 1 | 0.134 | 0.0172 |
| **N=500 P=10000** | **PSR** | 0.122 | 0.222 | 0.486 | 0.608 | 0.876 | 0.984 | 0.998 | 1 | 0.15 | 0.0226 |
|  | **PSW** | 0.114 | 0.246 | 0.458 | 0.542 | 0.774 | 0.902 | 0.968 | 0.998 | 0.408 | 0.0561 |
|  | **PSU** | 0.132 | 0.288 | 0.552 | 0.67 | 0.89 | 0.988 | 1 | 1 | 0.22 | 0.0315 |
|  | **COV** | 0.194 | 0.392 | 0.684 | 0.764 | 0.938 | 0.994 | 1 | 1 | 0.16 | 0.0223 |

* TPR measures the true positive rate towards each true mediator (M_1_-M_8_); FP is the false positive number and FDR is the false discovery rate (= FP/TP, where TP is the total positive numbers). All the indicators are the average over the 500 simulation repeats.

**Supplementary Table 6.** TPR, FP and FDR for the eight true mediators (M_1_-M_8_) $(\alpha=0.4t, \beta=0.6t$）

| **Sample & Dimension** | **Methods** | **TPR** | | | | | | | | **FP** | **FDR** |
| --- | --- | --- | --- | --- | --- | --- | --- | --- | --- | --- | --- |
|  |  | **M_1_**  **(αβ=0.0600)** | **M_2_**  **(αβ=0.0864)** | **M_3_**  **(αβ=0.1350)** | **M_4_**  **(αβ=0.1536)** | **M_5_**  **(αβ=0.2400)** | **M_6_**  **(αβ=0.3456)** | **M_7_**  **(αβ=0.5400)** | **M_8_**  **(αβ=0.9600)** |  |  |
| **N=300**  **P=1000** | **PSR** | 0.032 | 0.056 | 0.172 | 0.172 | 0.388 | 0.536 | 0.818 | 0.986 | 0.07 | 0.0141 |
|  | **PSW** | 0.062 | 0.108 | 0.222 | 0.192 | 0.382 | 0.506 | 0.718 | 0.888 | 0.276 | 0.0538 |
|  | **PSU** | 0.05 | 0.074 | 0.188 | 0.196 | 0.414 | 0.536 | 0.822 | 0.988 | 0.152 | 0.0333 |
|  | **COV** | 0.096 | 0.146 | 0.296 | 0.32 | 0.572 | 0.704 | 0.896 | 0.998 | 0.08 | 0.0146 |
| **N=300**  **P=10000** | **PSR** | 0.014 | 0.026 | 0.074 | 0.082 | 0.26 | 0.468 | 0.748 | 0.97 | 0.062 | 0.0157 |
|  | **PSW** | 0.014 | 0.05 | 0.108 | 0.116 | 0.258 | 0.468 | 0.692 | 0.896 | 0.384 | 0.0816 |
|  | **PSU** | 0.014 | 0.036 | 0.084 | 0.1 | 0.294 | 0.52 | 0.78 | 0.976 | 0.168 | 0.0416 |
|  | **COV** | 0.018 | 0.078 | 0.186 | 0.208 | 0.454 | 0.642 | 0.86 | 0.992 | 0.082 | 0.019 |
| **N=500**  **P=1000** | **PSR** | 0.138 | 0.298 | 0.484 | 0.508 | 0.71 | 0.918 | 0.988 | 1 | 0.128 | 0.0201 |
|  | **PSW** | 0.15 | 0.254 | 0.408 | 0.438 | 0.6 | 0.762 | 0.88 | 0.976 | 0.42 | 0.0661 |
|  | **PSU** | 0.146 | 0.29 | 0.48 | 0.498 | 0.708 | 0.91 | 0.98 | 1 | 0.266 | 0.0422 |
|  | **COV** | 0.232 | 0.388 | 0.604 | 0.638 | 0.82 | 0.956 | 0.994 | 1 | 0.122 | 0.018 |
| **N=500 P=10000** | **PSR** | 0.07 | 0.126 | 0.3 | 0.426 | 0.702 | 0.866 | 0.972 | 1 | 0.108 | 0.187 |
|  | **PSW** | 0.058 | 0.138 | 0.262 | 0.348 | 0.534 | 0.718 | 0.868 | 0.976 | 0.378 | 0.0635 |
|  | **PSU** | 0.072 | 0.156 | 0.3 | 0.422 | 0.692 | 0.874 | 0.974 | 1 | 0.206 | 0.0341 |
|  | **COV** | 0.134 | 0.278 | 0.482 | 0.584 | 0.796 | 0.918 | 0.986 | 1 | 0.106 | 0.0169 |

* TPR measures the true positive rate towards each true mediator (M_1_-M_8_); FP is the false positive number and FDR is the false discovery rate (= FP/TP, where TP is the total positive numbers). All the indicators are the average over the 500 simulation repeats.
